# TiO_2_-Mediated Photocatalytic Mineralization of a Non-Ionic Detergent: Comparison and Combination with Other Advanced Oxidation Procedures

**DOI:** 10.3390/ma8010231

**Published:** 2015-01-14

**Authors:** Péter Hegedűs, Erzsébet Szabó-Bárdos, Ottó Horváth, Krisztián Horváth, Péter Hajós

**Affiliations:** 1 Department of General and Inorganic Chemistry, Institute of Chemistry, University of Pannonia, P.O.Box 158, 8201 Veszprém, Hungary; E-Mails: peter.hegedus89@gmail.com (P.H.); bardos@vegic.uni-pannon.hu (E.S.-B.); 2 Department of Analytical Chemistry, Institute of Chemistry, University of Pannonia, P.O.Box 158, 8201 Veszprém, Hungary; E-Mails: raksi@almos.uni-pannon.hu (K.H.); hajosp@almos.uni-pannon.hu (P.H.)

**Keywords:** nonionic surfactant, advanced oxidation process, titanium dioxide, photocatalytic degradation, UHPLC, ozonation, persulfate

## Abstract

Triton X-100 is one of the most widely-applied man-made non-ionic surfactants. This detergent can hardly be degraded by biological treatment. Hence, a more efficient degradation method is indispensable for the total mineralization of this pollutant. Application of heterogeneous photocatalysis based on a TiO_2_ suspension is a possible solution. Its efficiency may be improved by the addition of various reagents. We have thoroughly examined the photocatalytic degradation of Triton X-100 under various circumstances. For comparison, the efficiencies of ozonation and treatment with peroxydisulfate were also determined under the same conditions. Besides, the combination of these advanced oxidation procedures (AOPs) were also studied. The mineralization of this surfactant was monitored by following the TOC and pH values, as well as the absorption and emission spectra of the reaction mixture. An ultra-high-performance liquid chromatography (UHPLC) method was developed and optimized for monitoring the degradation of Triton X-100. Intermediates were also detected by GC-MS analysis and followed during the photocatalysis, contributing to the elucidation of the degradation mechanism. This non-ionic surfactant could be efficiently degraded by TiO_2_-mediated heterogeneous photocatalysis. However, surprisingly, its combination with the AOPs applied in this study did not enhance the rate of the mineralization. Moreover, the presence of persulfate hindered the photocatalytic degradation.

## 1. Introduction

In our natural waters, artificial detergents can threaten self-cleaning processes, such as oxygen/carbon dioxide exchange and sedimentation of floating particles. As pollutants, through the channel systems, they can get into our environment and may solubilize various water-insoluble pesticides, polyaromatic hydrocarbons and other types of organic compounds [1,2,3,4]. These, along with the surfactants themselves, may be toxic for microorganisms. A considerable part of synthetic detergents is represented by non-ionic surfactants. They are more stable than ionic tensides and not sensitive to the pH and electrolytes of the aqueous systems in which they are involved. Currently, the non-ionic surfactants of the alkylphenyl polyethoxylate type (Triton X-*n*) [5], where *n* can be within the range of 3–40, are the most widely used at the industrial scale. They are applied in household and industrial cleaning agents, paints and coatings, as well as utilized in the dye and textile industries as detergents, emulsifiers, wetting agents, solubilizers and dispersants [6,7,8]. Triton X-100 with an average *n* ≈ 9.5 is one of the most widespread man-made nonionic surfactants. Besides the hydrophilic polyethylene oxide chain, it also contains a hydrophobic octylphenyl group.

In most large sewage farms, the degradation of organic pollutants takes place in biological systems following physical preparation steps. However, Triton X-100 can hardly be degraded by biological treatment under anaerobic conditions, and even in aerobic systems, it can be just partly mineralized in this way [6,9,10]. Thus, as a consequence of the incomplete degradation, it can reach from the sewage farms to natural waters, damaging the various living organisms there [11,12]. It may destroy the cell membranes [13,14,15] and hinder the function of the peripheral nervous system [16]. Thus, they are potentially hazardous with respect to the contamination of ground water and drinking water supplies [17].

Hence, a more efficient degradation method is indispensable for the total mineralization of this dangerous surfactant. Oxidation of the polyoxyethylene chain was carried out by using Ag^III^(H_2_IO_6_)(H_2_O)_2_ (DPA) [18]. The primary products of this process were acetaldehyde and 4-(1,1,3,3-tetramethylbutyl)phenol. Besides, various advanced oxidation processes (AOPs) were also applied for the degradation of this detergent. Hydrated electron produced by pulse radiolysis, in the presence of *t*-butanol, did not prove to be efficient enough [7,19]. Its reaction with various scavengers, such as O_2_ and N_2_O, led to the formation of hydroxyl radicals, which oxidized the aromatic ring of the tenside via hydrogen abstraction.

Heterogeneous photocatalysis based on titanium dioxide was also applied, but giving contradictory results. At similar a concentration of surfactant, the optimum catalyst concentrations found deviate by one order of magnitude [20,21]. Modification of the photocatalyst with SiO_2_ or Pt increased its activity by a factor of two [22]. The addition of H_2_O_2_ or, especially, K_2_S_2_O_8_ also increased the degradation efficiency [21,23] of the photocatalytic procedure. Different sources of UV light were used in these studies. Besides, various 4-alkylphenols were also degraded by heterogeneous photocatalysis, but utilizing visible light [24,25]. The hydrophobic part of these complexes is very similar to that of the components of Triton X-100. Intermediates were detected in these studies by the LC-ESI-MS and GC-MS methods [23,24], but the time dependence of their concentration was not followed.

Our current investigations indicated TiO_2_-mediated photocatalysis to be successfully applicable to the mineralization of various ionic detergents [26,27,28] and amino acids [29]; besides, its combination with ozonation resulted in a synergistic effect [30,31]. On the basis of the earlier, partly inconsistent observations, our present paper deals with the oxidation and mineralization of Triton X-100 under various circumstances, focusing on the heterogeneous photocatalysis, in order to get more insight into the degradation mechanism. For comparison, the efficiencies of ozonation and treatment with peroxydisulfate were also determined under the same conditions. Besides, the effects of the combination of these advanced oxidation procedures (AOPs) were also investigated.

## 2. Results and Discussion

### 2.1. TiO_2_/UV/Air System

**Figure 1:**
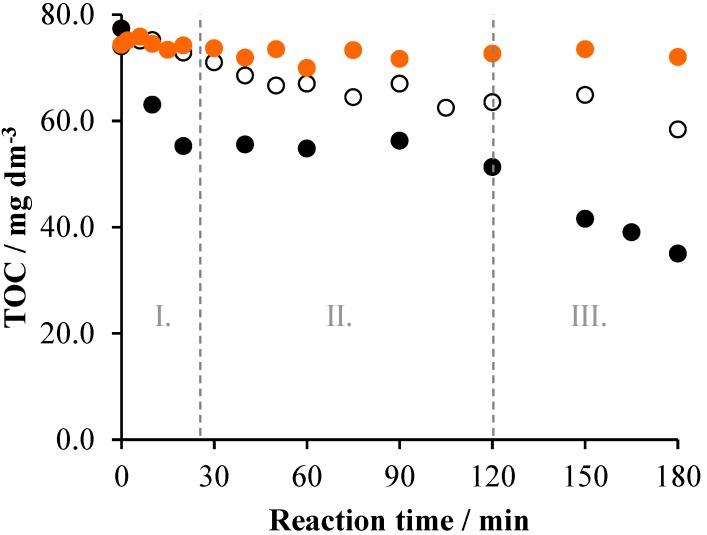
The change of the TOC as a function of time under various conditions in the system containing 2 × 10^−4^ mol·dm^−3^ Triton X-100 and 1 g·dm^−3^ TiO_2_: air (•), air/UV (○) and air/UV/TiO_2_ (•).

Before measuring the mineralization of Triton X-100 by heterogeneous photocatalysis, it was investigated in the absence of the TiO_2_ photocatalyst, as well as without irradiation. As Figure 1 indicates, no decrease of the TOC was observed during a 3-h stirring with air bubbling. Under the same conditions, but irradiated at λ_ir_ > 300 nm, however, a moderate mineralization took place: 21%. The initial rate of the TOC decrease was 0.26 mg·dm^−3^·min^−1^. This result indicates that also a direct photolysis of the surfactant can happen in the aerated system, which may be attributed to the excitation of the tenside due to the slight overlap of its absorption spectrum and the emission spectrum of the UV light source applied. In the presence of 1 g·dm^−3^ TiO_2_ photocatalyst, the initial rate and the extent of mineralization significantly increased (to 1.75 mg·dm^−3^·min^−1^ and 55%, respectively). Besides, the TOC *versus* reaction time function can be divided into three unambiguously distinguishable sections (Figure 1).

In the first 20 min, the TOC decreased quickly, from 74 mg·dm^−3^ to 58.4 mg·dm^−3^. However, in the subsequent 100-min period, practically no change of the TOC took place. This result suggests that only intermediates (oxidized derivatives) were formed during this period, without any mineralization. In the last 60 min, the TOC decreased again, although at a lower rate (0.67 mg·dm^−3^·min^−1^) than initially. In this period, mineralization of the intermediates formed in the initial stage took place. The pH of the reaction mixture changed from 5.9 to 3.2 during the 3-h irradiation.

**Figure 2:**
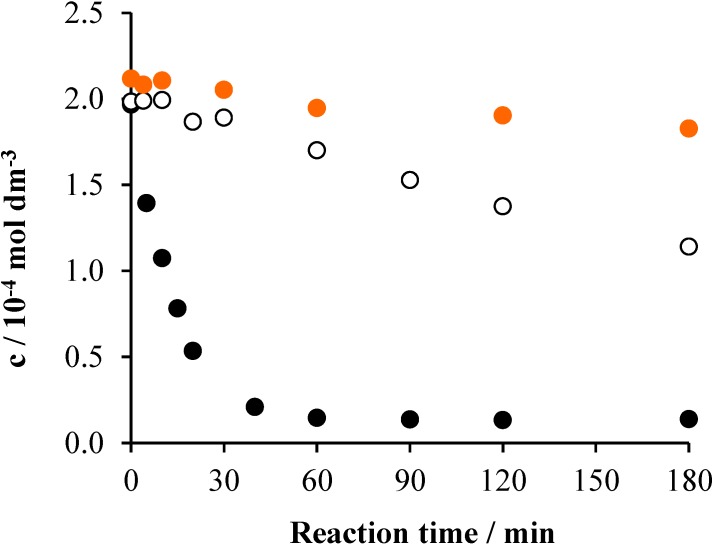
The change of the Triton X-100 concentration as a function of the reaction time under various conditions in the system containing 2 × 10^−4^ mol·dm^−3^ Triton X-100 and 1 g·dm^−3^ TiO_2_: air (•), air/UV (○) and air/UV/TiO_2_ (•).

The actual concentration of the surfactant was followed by UHPLC during the reaction time. The results are shown in Figure 2.

In accordance with the TOC *versus* time plots (Figure 1), no appreciable change was observed without irradiation, while in the irradiated system (in the absence of the catalyst), a 42.5% decrease took place at an initial rate of 6 × 10^−7^ mol·dm^−3^·min^−1^. This result indicates that this surfactant, even if to a moderate extent, can be transformed under natural conditions, upon solar irradiation. In the presence of TiO_2_ photocatalyst, the concentration of the starting tenside diminished below the detection limit within the first hour of irradiation (Figure 2). The initial rate of its disappearance was 10^−5^ mol·dm^−3^·min^−1^, unambiguously demonstrating an efficient transformation of this pollutant. The decrease of the detergent concentration in the photocatalytic degradation obeyed first-order kinetics (Figure S1), in accordance with earlier observations [20,21,23]. However, instead of the apparent rate constants, the initial rates were used for comparison, because the kinetics of the decay was not unambiguously first order in the case of the thermal reactions.

**Figure 3:**
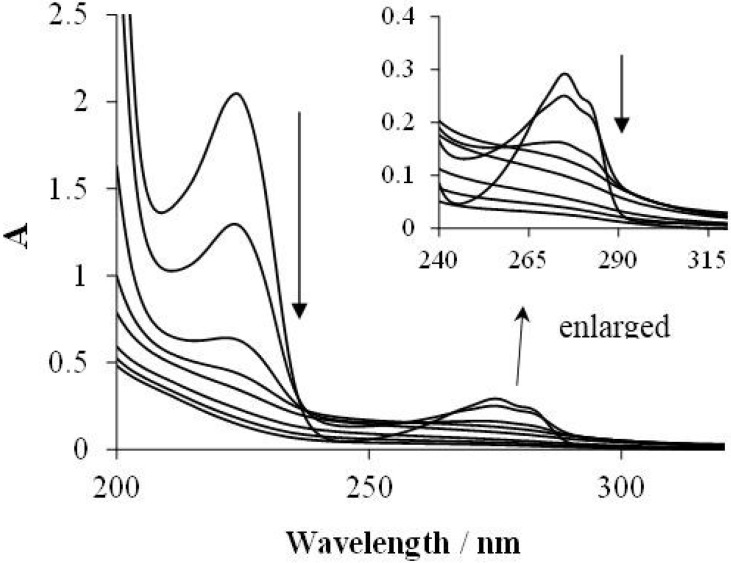
The change of the absorption spectrum (after removal of the suspended TiO_2_) during the photocatalysis in the aerated system containing 2 × 10^−4^ mol·dm^−3^ Triton X-100 and 1 g·dm^−3^ catalyst (ℓ = 1 cm).

Furthermore, the change of the absorption spectrum displayed the degradation of Triton X-100 in the latter system (containing TiO_2_). As Figure 3 shows, the model compound displays two intense bands in the 200–350-nm range: one at 210–240 nm and another one at 250–290 nm. The latter band can be assigned to the π→π* transition characteristic of the aromatic system. During the degradation process, the absorbances of both bands gradually decreased.

**Figure 4:**
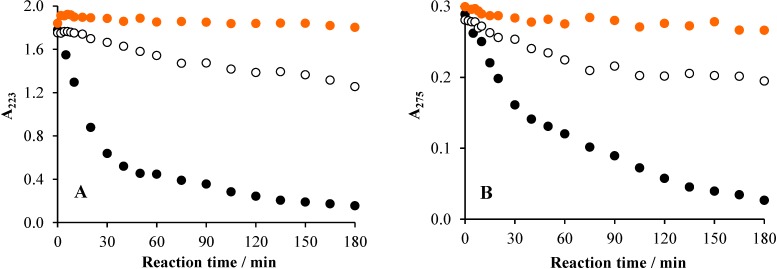
The change of the absorbance at 223 nm (**A**) and 275 nm (**B**) as a function of the reaction time under various conditions in the system containing 2 × 10^−4^ mol·dm^−3^ Triton X-100 and 1 g·dm^−3^ TiO_2_ (ℓ = 1 cm): air (•), air/UV (○) and air/UV/TiO_2_ (•).

Surprisingly, no shift of the absorption bands was observed, which suggests that no hydroxylation precedes the ring opening. Figure 4 displays the absorbance *versus* reaction time plots under various conditions at the characteristic wavelengths (223 and 275 nm).

**Figure 5:**
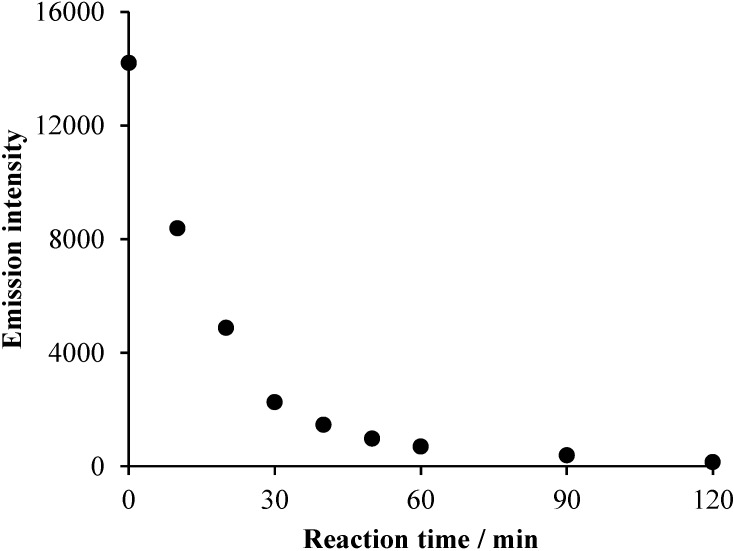
The change of the emission intensity (after removal of the suspended TiO_2_) during the photocatalysis in the aerated system containing 2 × 10^−4^ mol·dm^−3^ Triton X-100 and 1 g·dm^−3^ catalyst (ℓ = 1 cm, λ_ex_ =277 nm, λ_em_ =302 nm).

In accordance with the concentration *versus* time plots, negligible change was observed without irradiation, while direct photolysis caused a moderate, but continuous, decrease of the absorbance at 223 nm. However, at 275 nm in the 90–120-min range, no significant change can be observed, indicating that at this wavelength, the relatively stable intermediates formed display absorption. In the case of the photocatalytic degradation, much faster and continuous decreases of absorbance are shown, but at 223 nm, the rate of the absorption change is significantly higher than at 275 nm. This phenomenon, in accordance with the conclusion regarding the direct photolysis, suggests that the absorption at the latter (longer) wavelength can be attributed to more stable intermediates than those absorbing at 223 nm. Besides, the change of the intensity of emission originating from the aromatic moiety of the molecules indicates that, already in the early stage of the photocatalytic degradation, a significant part of the benzene rings was destroyed (Figure 5), in accordance with the observations regarding alkylphenols in very similar systems [24]. The decay of this emission proved also to be of first-order kinetics (Figure S2). Similarly to the absorption spectra, no band-shift was observed during the irradiation, indicating that no significant hydroxylation of the aromatic ring occurred. Notably, in the argon-saturated reaction mixture, TiO_2_-based photocatalysis cannot lead to the cleavage of the aromatic ring, and only emissive hydroxylated derivatives are formed [26,28].

### 2.2. Effects of Na_2_S_2_O_8_

**Figure 6:**
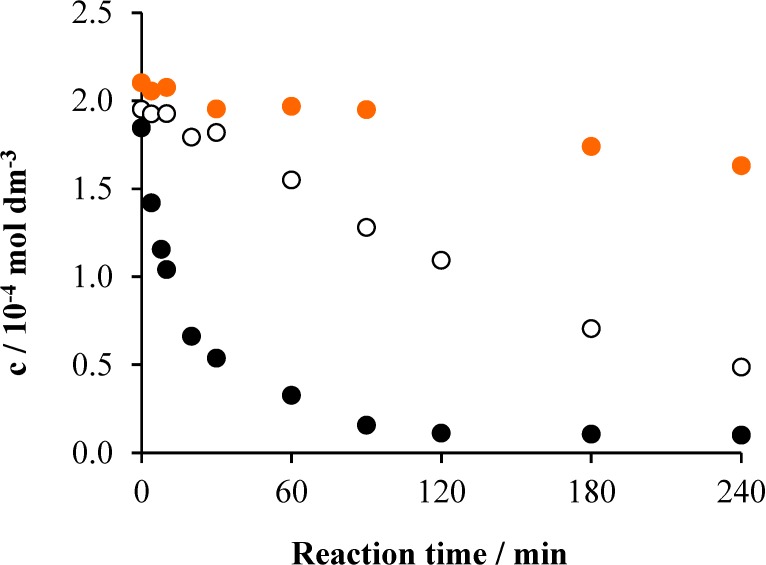
The change of the Triton X-100 concentration as a function of the reaction time under various conditions in the system containing 2 × 10^−4^ mol·dm^−3^ Triton X-100 and 1 × 10^−3^ mol·dm^−3^ Na_2_S_2_O_8_: air (•), air/UV (○) and air/UV/TiO_2_ (1 g dm^−3^) (•).

Regarding the effects of oxidative additives, firstly, the application of peroxydisulfate (or persulfate) was investigated: in the dark (Na_2_S_2_O_8_/air), irradiated (Na_2_S_2_O_8_/air/UV) and combined with heterogeneous photocatalysis (Na_2_S_2_O_8_/air/UV/TiO_2_). Since persulfate is an efficient oxidizing agent in thermal processes, a 22% decrease of the surfactant concentration was observed after a four-hour reaction time, with a 2 × 10^−7^ mol·dm^−3^·min^−1^ initial rate (Figure 6). No change of the pH accompanied this process.

Upon irradiation of this system, under the same conditions as in the dark, a considerably higher concentration decrease (76%) was observed and, accordingly, a four-times higher initial rate (8 × 10^−7^ mol·dm^−3^·min^−1^). Notably, this concentration decrease is about the sum of those observed for the air/UV and Na_2_S_2_O_8_/air systems after 4 h (54% + 22%). The pH significantly changed (from 5.4 to 3), which may be the consequence of H^+^ formation in the reactions of the sulfate radical-anion (SO_4_^•−^) generated (Equations (1)–(5)) [32]:

S_2_O_8_^2−^ + hν → 2 SO_4_^•−^ (1)

SO_4_^•−^ + H_2_O → HO^•^ + SO_4_^2−^ + H^+^ (2)

SO_4_^•−^ + HO^−^ → HO^•^ + SO_4_^2−^ (3)

S_2_O_8_^2−^ + H^+^ → HS_2_O_8_^−^ (4)

HS_2_O_8_^−^ → SO_4_^•−^ + SO_4_^2−^ + H^+^ (5)

Since the photoinduced dissociation of persulfate (Equation (1)), however, needs excitation at wavelengths shorter than 310 nm, our light source hardly promoted this reaction. The thermal reactions between persulfate and the excited surfactant, the formation of which is indicated by the results of the direct photolysis (see Section 2.1.), can give a more significant contribution to the acidification. In the system containing photocatalyst, also, the concentration of Triton X-100 decreased below the detection limit within about 90 min (Figure 6). The initial rate was 10^−5^ mol·dm^−3^·min^−1^, which agrees with that observed for the photocatalytic degradation in the absence of persulfate. This result indicates that, deviating from earlier observations [21,23], the addition of persulfate did not increase the efficiency of the degradation of Triton X-100. The different light sources used are the main reason for this deviation. While in this study, the light tube emitted at 350 nm, in the previous works, irradiations at much shorter wavelengths were applied, e.g., 254 nm in [23]. Thus, Reaction (1) efficiently took place in those experiments, while the energy of our light source was not enough for that process. Moreover, in our case, the addition of persulfate apparently hindered the photocatalytic process, as the corresponding plots in Figure 2 and Figure 6 demonstrate. This phenomenon may be attributed to the occupation of the active sites on the surface of the catalyst by the persulfate ions or sulfate ions formed from the previous ones. Due to the more effective oxidative degradation in the presence of TiO_2_, the acidification is one order of magnitude higher than in the system without photocatalyst; the pH changed from 5.4 to 2.

**Figure 7:**
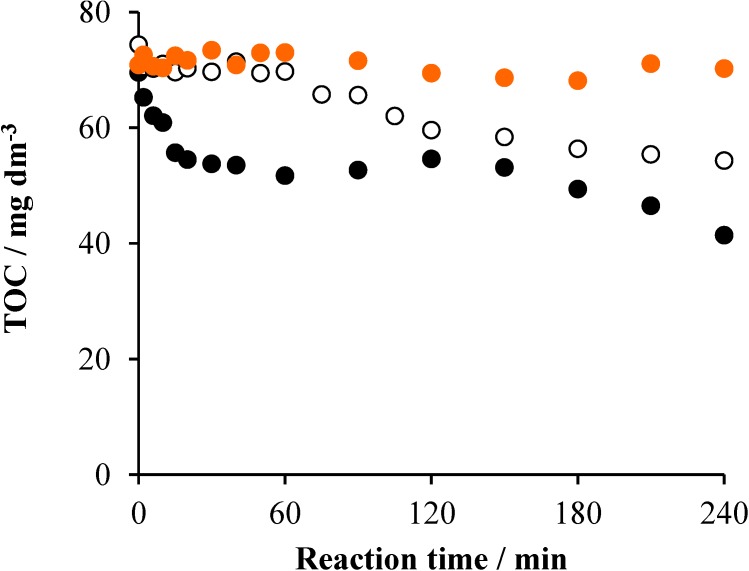
The change of TOC as a function of the reaction time under various conditions in the system containing 2 × 10^−4^ mol·dm^−3^ Triton X-100 and 10^−3^ mol·dm^−3^ Na_2_S_2_O_8_: air (•), air/UV (○), air/UV/TiO_2_ (1 g dm^−3^) (•).

Comparing the mineralization efficiencies (*i.e.*, the TOC *versus* time plots), the tendencies are similar to those observed for the decrease of the surfactant concentration. As Figure 7 displays, no change of the TOC happened in the dark, while a moderate, but continuous, decrease took place in the irradiated system without catalyst, with a 0.01 mol·dm^−3^·min^−1^ initial rate and 27% mineralization (within 4 h). The presence of the photocatalyst increased the initial rate by two orders of magnitude (1.07 mol·dm^−3^·min^−1^) and the mineralization to 40%. However, these values are considerably lower than the corresponding ones observed for the heterogeneous photocatalysis in the absence of persulfate (55% and 1.75 mol·dm^−3^·min^−1^), confirming the hindering effect also observed for the decrease of the surfactant concentration. This contrast with the earlier observations [21,23] may be attributed to the different conditions; e.g., a two orders of magnitude lower concentration of TiO_2_ was applied in those experiments [21] than in the present work; also, the intensities and the emission spectra of the light sources were considerably different, as indicated above. Besides, in our study, the concentration of the detergent was about one order of magnitude higher than those in the previous works.

### 2.3. Effects of Ozonation

**Figure 8:**
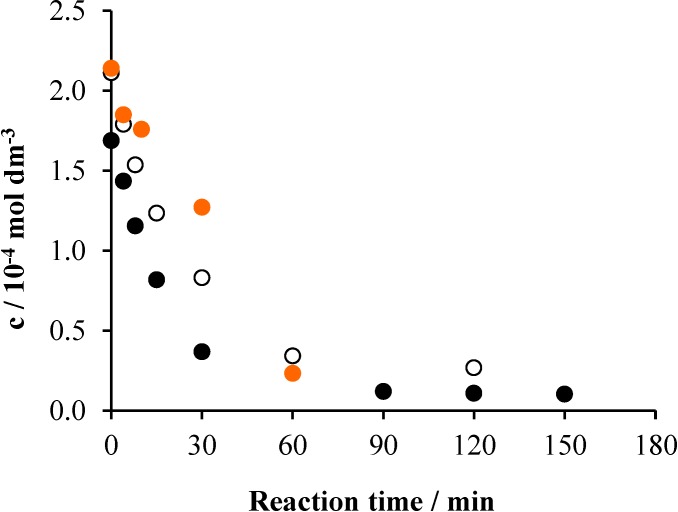
The change of the Triton X-100 concentration as a function of the reaction time under various conditions in the system containing 2 × 10^−4^ mol·dm^−3^ Triton X-100 and ozonated at 3.5 × 10^−4^ mol·dm^−3^·min^−1^ rate: air (•), air/UV (○) and air/UV/TiO_2_ (1 g·dm^−3^) (•).

Ozone was generated in the air stream bubbled through the reaction mixture at a 40-dm^3^·h^−1^ rate. The input ozone current was 3.5 × 10^−4^ mol·dm^−3^·min^−1^. Also in this case, experiments under three different conditions were carried out: in the dark (O_3_/air), irradiated (O_3_/air/UV) and combined with heterogeneous photocatalysis (O_3_/air/UV/TiO_2_). Like persulfate, ozone is also an efficient oxidizing agent in thermal processes. Accordingly, the concentration of the surfactant decreased below the detection limit within an hour, with an initial rate of 2 × 10^−6^ mol·dm^−3^·min^−1^ (Figure 8). Irradiation and photocatalysis accelerated the transformation of Triton X-100; the initial rate tripled in in these cases (6 × 10^−6^ mol·dm^−3^·min^−1^).

**Figure 9:**
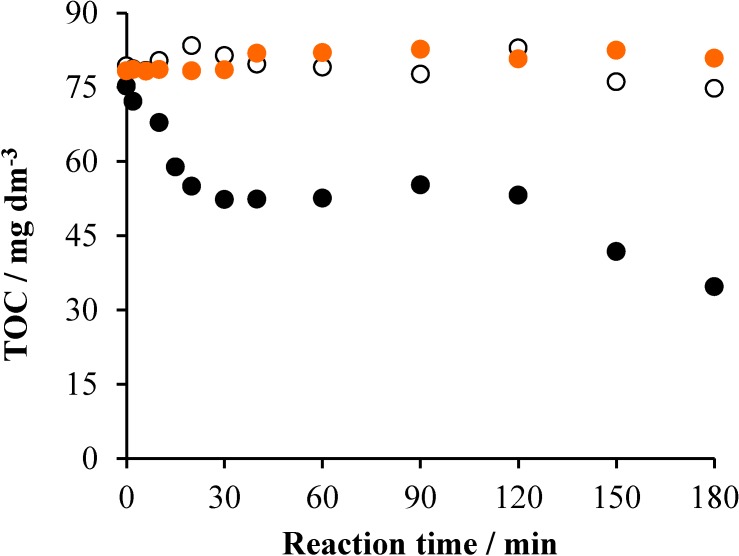
The change of the TOC as a function of the reaction time under various conditions in the system containing 2 × 10^−4^ mol·dm^−3^ Triton X-100 and ozonated at 3.5 × 10^−4^ mol·dm^−3^·min^−1^ rate: air (•), air/UV (○) and air/UV/TiO_2_ (1 g dm^−3^) (•).

Regarding the mineralization of Triton X-100 by ozonation, no change of the TOC was observed without irradiation and, deviating from the case of persulfate, the concentration of the total organic carbon did not appreciably decrease, even in the irradiated system (O_3_/air/UV), as shown in Figure 9.

These data, compared to those in Figure 8, indicate that the considerable transformation (oxidation) of the surfactant in the O_3_/air and O_3_/air/UV systems resulted in the formation of intermediates, which did not mineralize at all during the 3-h reaction time. The combination with photocatalysis led to the same mineralization efficiency (54%), as in the case of the air/UV/TiO_2_ system, and to an initial rate (1.44 mg·dm^−3^·min^−1^), which is just slightly lower than the corresponding value measured without ozonation. These data suggest that ozonation, deviating from our observations regarding ionic surfactants [30,31], does not increase the efficiency of the photocatalytic mineralization of Triton X-100. However, contrary to persulfate, O_3_ does not show any significant hindering effect either, probably because it cannot occupy the active sites on the surface of the catalyst.

### 2.4. Effects of the Initial pH

**Figure 10:**
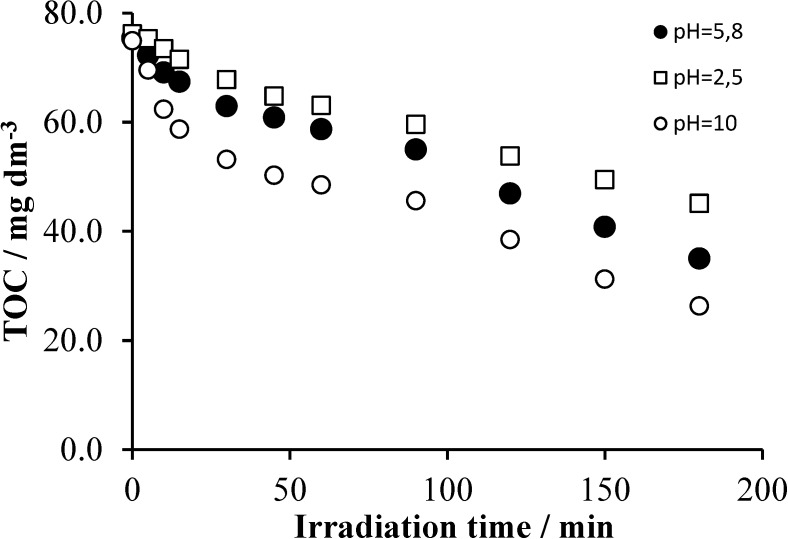
The change of the TOC as a function of the irradiation time at various pH values in the aerated system containing 2 × 10^−4^ mol·dm^−3^ Triton X-100 and 1 g·dm^−3^ TiO_2_.

According to recent observations, pH considerably affects the rate of the primary oxidation of Triton X-100 in the TiO_2_-based photocatalytic degradation [23]. Those results indicated that the pH values where the catalyst surface is close to neutral are favorable for the reaction, promoting the adsorption of the non-ionic surfactant. Thus, pH values near to that of the isoelectric point (IEP) of titania (6.8 [33]) are most suitable in this respect. This conception is in accordance with the Langmuir–Hinshelwood model, and the primary oxidation reaction of Triton X-100 was found to obey [23]. However, the mineralization of this detergent, *i.e.*, the decrease of the TOC of its solution, does not necessarily follow this tendency. As Figure 10 displays, the pH effect on the mineralization rate of Triton X-100 is different from that regarding its primary oxidation step.

In this case, apparently, the increase of pH in the range studied enhanced the rate of mineralization. These results indicate that degradation of the intermediates formed in the primary processes is promoted by the hydroxide ions. Since a strong acidification was observed during the photocatalytic degradation of Triton X-100 (see Section 2.2.), an increase of pH may enhance the driving force of the corresponding oxidation reactions of mineralization. On the basis of the plots in Figure 10, the most significant effect of pH can be observed in the first 25–30-min period of irradiation, where the rate of the TOC change is the highest at each pH value. This phenomenon suggests that in this period, mostly the species formed by the cleavage of the last member of the polyethoxylate chain are mineralized, obviously faster than those (bigger ones) formed in the fragmentation along the whole chain. Oxidation of these species takes place via the formation of carboxylic acids, the deprotonation of which generates anions not being favored with respect to the adsorption on the negatively-charged surface at pH > 7. Hence, the promoting effect of the increased pH may be attributed to the redox reactions of the intermediates containing one or two carbon atoms, in the solution phase.

### 2.5. Mechanistic Considerations; Intermediates

**Figure 11:**
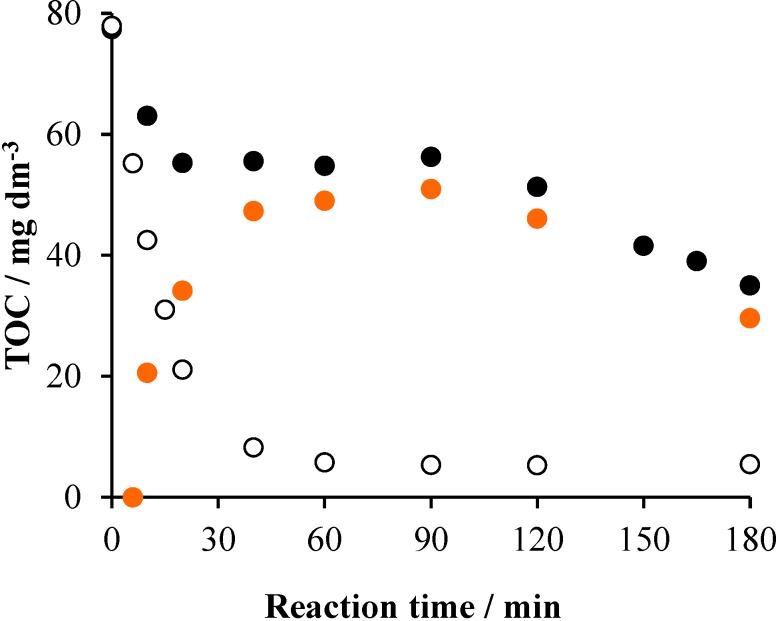
The change of the TOC as a function of time in the system containing 2 × 10^−4^ mol·dm^−3^ Triton X-100 and 1 g·dm^−3^ TiO_2_: for the whole system (•), for the unreacted surfactant (○) and for the intermediates (•).

In order to get insight into the mechanism of the photocatalytic degradation and mineralization of Triton X-100, also some data regarding the intermediates formed were determined. Although the measured TOC values concern the whole reaction mixture, those belonging to the intermediates can also be determined. This is the difference of the TOC belonging to the overall system and that corresponding to the unreacted surfactant. The latter one can be calculated from the actual concentration of Triton X-100 measured by UHPLC. Figure 11 displays these TOC *versus* time plots obtained for the air/UV/TiO_2_ system.

In the first 60 min, the TOC representing the intermediates steeply increased, accompanied by the similarly fast and finally total disappearance of the starting surfactant. In the 60–120-min period, the TOC of the intermediates hardly changed, indicating that mostly their oxidation/oxygenation and cleavage took place. Subsequently, their mineralization sped up due to the oxidation of the short ethoxy chains.

#### 2.5.1. UHPLC Measurements

**Figure 12:**
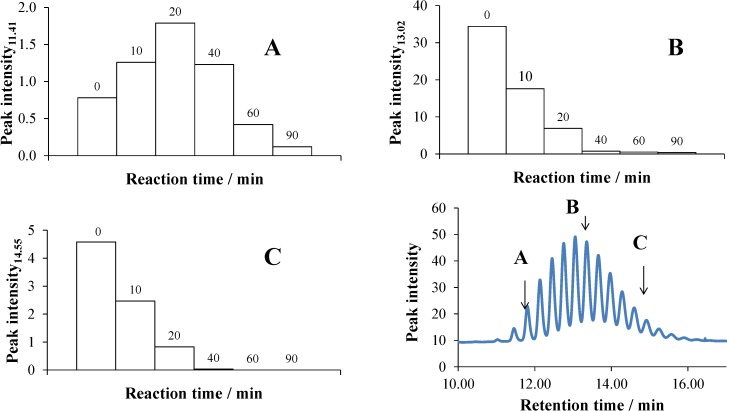
The change of the peak intensity as a function of the reaction time from the UHPLC chromatograms obtained during the photocatalysis of the system containing 2 × 10^−4^ mol·dm^−3^ Triton X-100 and 1 g·dm^−3^ TiO_2_: at a retention time of 11.41 min (**A**), 13.02 min (**B**) and 14.55 min (**C**).

Since the UHPLC chromatogram of Triton X-100 consists of several peaks corresponding to the components with various lengths of ethoxy chains (Figure 12), its time-dependent change demonstrates well how the concentrations of these components are affected during the photocatalysis. The retention time of these non-ionic surfactant components is in strong correlation with the length of the ethoxy chain, *i.e.*, the number of the ethoxy groups (*n*); a longer chain corresponds to a higher *n*. The column charts in Figure 12A–C demonstrate well how the representative peaks (and, thus, the concentrations) of the components of different lengths change as a function of time. The peak intensity of the shorter chain components (with retention times of 10.4–11.76 min) increased in the first 20 min of the reaction, then decreased (Figure 12A). The peak intensity of the components with a 12.09–14.24-minute retention time (Figure 12B) decreased slower than that belonging to the long-chain components (with retention times of 14.55–15.87 min, Figure 12C). The concentration of the latter group diminished below the detection limit already within 40 min.

**Figure 13:**
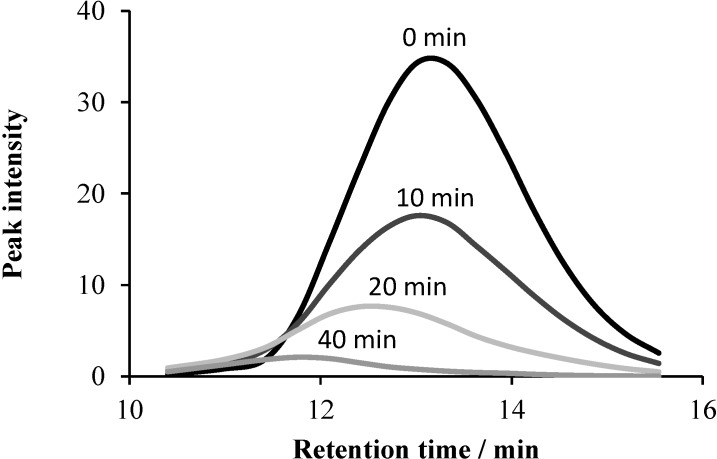
The peak intensity as a function of the retention time, taken from the UHPLC chromatograms during the photocatalysis of the system containing 2 × 10^−4^ mol·dm^−3^ Triton X-100 and 1 g·dm^−3^ TiO_2_, after 0, 10, 20 and 40 min of reaction time.

Analyzing the chromatograms, the envelopes of the peak intensities belonging to different retention times after various reaction times were also plotted (Figure 13). It is clearly seen that the retention time of the maximum peak intensity gradually decreased during the photocatalytic process, *i.e.*, the components with longer chains degraded faster, also in accordance with the column charts in Figure 12.

These results suggest that in the heterogeneous photocatalytic degradation of Triton X-100, under our experimental conditions, the attack by the photogenerated hydroxyl radicals is favored at the ethoxy side-chain. This conclusion is confirmed by the change of the absorption spectrum (Figure 3); no shift of the longer wavelength (275 nm) band was observed, *i.e.*, no hydroxylation of the aromatic ring took place. After the total disappearance of the starting surfactant (after *ca.* 60 min), an appreciable absorbance of the 275-nm band remained, which indicates that also intermediates with an aromatic ring were formed during the first hour. Further irradiation led to the cleavage of the aromatic ring, giving intermediates that do not absorb in the longer wavelength range. In accordance with our observation, intermediates with a hydroxylated aromatic ring were not detected during the degradation of this non-ionic surfactant and similar alkylphenol ethoxylates in other advanced oxidation procedures [34,35,36,37,38]. The lack of the formation of intermediates with a hydroxylated aromatic ring deviates from our earlier observations regarding the photocatalytic degradation of benzenesulfonate and phenylalanine [28,29]. These results may be related to the facts that the efficiency for the photocatalytic mineralization of the latter compounds was increased by ozonation in a synergistic way, while in the case of Triton X-100, O_3_ did not accelerate the TiO_2_-mediated degradation.

#### 2.5.2. GC-MS Measurements

The UHPLC analysis did not give any information regarding the intermediates, the structures of which significantly deviate from those of the components of Triton X-100. Thus, in order to detect such intermediates also, GC-MS measurements were also carried out after the solid-phase extraction as described in the Experimental Section. Although this method is suitable only to the detection of species of lower molecular weight, the tendencies observed for those can be generalized for the transformation of the bigger components of Triton X-100.

**Figure 14:**
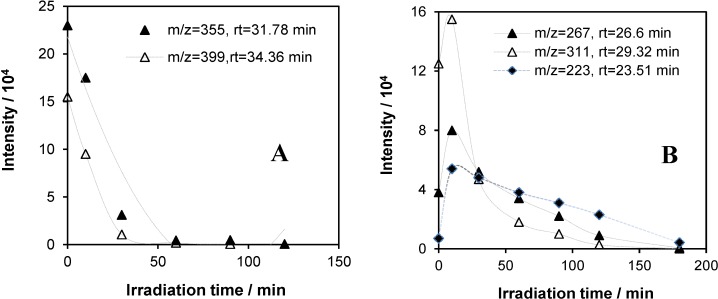
Intensity *vs.* irradiation time plots for the most abundant fragment ion of the starting components of Triton X-100 with molecular weights of 470 (*m*/*z* = 399), 426 (*m*/*z* = 355) (**A**) and 382 (*m*/*z* = 311), 338 (*m*/*z* = 267) and 294 (*m*/*z* = 223) (**B**) in the photocatalysis of the system containing 2 × 10^−4^ mol·dm^−3^ Triton X-100 and 1 g·dm^−3^ TiO_2_.

The following figures present the intensity *versus* irradiation time plots for the most abundant fragment ion of the species, which could be identified unambiguously or with high probability on the basis of their mass spectra and retention times (the ion chromatograms belonging to the different irradiation times and the mass spectra of typical components in the reaction mixture, identified by our GC-MS measurements, can be found in the Supplementary Material as Figures S3 and S4, respectively). Figure 14A shows the starting components of Triton X-100 with an ethoxylate number (*n*) of five and six, *i.e.*, with molecular weights of 426 and 470, respectively. The concentration of these components decreased from the very beginning of the irradiation and practically disappeared within 40 minutes. However, the concentration of the components with *n* = 2, 3 and 4, *i.e.*, with molecular weights of 294, 338 and 383, respectively, increased in the first 10 min, which was followed by a gradual decay. These results are in accordance with those of the UHPLC measurements, indicating that the fragmentation of the longer polyethoxylate chains of the starting components initially increased the concentration of those with shorter ones. Notably, the *m/z* value corresponding to the most abundant fragment ion (*i.e.*, the base peak) differs from the molecular weight (*i.e.*, the *m/z* value of the mother peak) by 71 in each case in Figure 14, indicating compounds of the same type of structure (*i.e.*, (CH_3_)_3_C-CH_2_-(CH_3_)_2_C-C_6_H_4_-O-(CH_2_CH_2_O)_n_-H), from which the fragment ion was formed by the loss of the pentyl (*i.e.*, (CH_3_)_3_C-CH_2_-) group.

**Figure 15:**
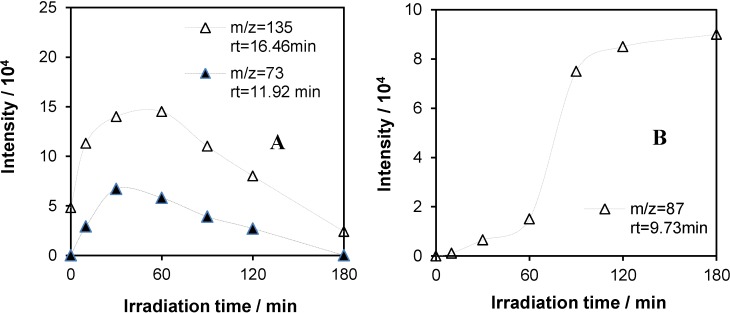
Intensity *vs.* irradiation time plots for the most abundant fragment ion of characteristic intermediates with molecular weights of 206 (*m*/*z* = 135), 148 (*m*/*z* = 73) (**A**) and 118 (*m*/*z* = 87) (**B**) in the photocatalysis of the system containing 2 × 10^−4^ mol·dm^−3^ Triton X-100 and 1 g·dm^−3^ TiO_2_.

The intensity *versus* irradiation time plots for the most abundant fragment ion of characteristic intermediates detected by GC-MS are shown in Figure 15. As can be seen, not only the molecular weights, but also the corresponding retention times are significantly smaller than those of the starting components (even if of lower *n* values). The maximum concentration of them belong to longer times; moreover, the smallest one (Figure 15B) is the most abundant intermediate detected by this method after a 180-min irradiation. As to the structure of these intermediates, *M* = 206 (*m*/*z* = 135, Figure 15A) could be unambiguously identified as 4-(1,1,3,3-tetramethylbutyl)phenol, *i.e.*, the alkylphenol (=octylphenol) part of the starting components of Triton X-100. This is the result of the total cleavage of the polyethoxylate chains without any oxidation of the rest of the original tenside molecules. In this case also, similarly to the mass spectra of the starting components, the *m/z* value of the base peak differs from that of the mother peak by 71, due to the loss of the same pentyl group.

The time dependence of the concentration of this intermediate indicates that it can be formed in a relatively early stage of the photocatalysis, obviously from the starting components with shorter ethoxylate chains and later also from those with longer chains. Thus, the decay of this intermediate lasts till the end of the 3-h irradiation period.

The other plot in Figure 15A can be assigned to HO(CH_2_CH_2_O)_2_CH_2_CHO (*M* = 148, the base peak of *m/z* = 73 belongs to the –CH_2_OCH_2_CHO fragment), which is clearly derived from the ethoxy chain of the starting molecules via fragmentation. Thus, it is a kind of complementary intermediate of the octylphenol part previously discussed. Both are the results of the same type of fragmentation. The formation of these intermediates is in full accordance with the results recently obtained by the LC-ESI-MS method [23].

Oxidation of these species led to other intermediates of various structures, the identification of which needs further investigations. As our UV (absorption and emission) spectral study indicated (see Section 2.1.), they involve ring-opened compounds also, in agreement with the results of GC-MS analysis regarding the photocatalysis of different alkylphenols [24]. Nevertheless, the intermediate for the most abundant fragment ion, of which the intensity *versus* time plot is given in Figure 15B, may be assigned as a short-chain (with a carbon number of five) hydroxy carboxylic acid or ester. Its complete oxidation (*i.e.*, total mineralization) would need an extended irradiation.

## 3. Experimental Section

### 3.1. Materials

In all experiments of this work, the titanium dioxide catalyst used was Degussa P25 (70% anatase, 30% rutile; with a surface area of 50 m^2^·g^−1^, Evonik-Degussa GmbH, Essen, Germany). The concentration of TiO_2_ was 1 g·dm^−3^. All other materials, such as Na_2_S_2_O_8_ (Merck KGaA, Darmstadt, Germany) and Triton X-100 (Alfa Aesar, Johnson Mattey Company, Ward Hill, MA, USA), were of reagent grade. Its concentration was 2 × 10^−4^ mol·dm^−3^ (=0.126 g·dm^−3^) in each degradation experiment. Compressed air was bubbled through the reaction mixtures from gas bottles, serving for both stirring and (with its O_2_ content) as an electron acceptor. O_3_ was produced by a LAB2B ozone generator and introduced in the same air stream. In all of these experiments, the ozone dosage was adjusted to 3.5 × 10^−4^ min^−1^. High purity water used as a solvent in this study was double distilled and then purified with a Milli-Q system. In order not to disturb the subsequent analyses, no buffer was used in the reaction mixtures to be irradiated.

### 3.2. Photochemical Experiments

**Figure 16:**
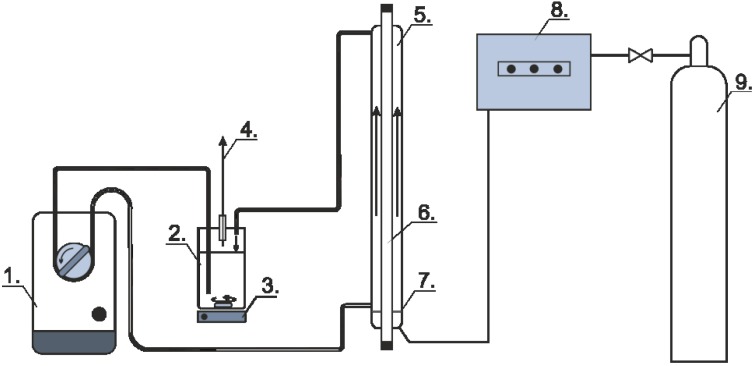
Sketch of the photocatalytic reactor with the auxiliary units: 1, peristaltic pump; 2, buffer vessel; 3, magnetic stirrer; 4, sampling port; 5, reactor (Pyrex vessel); 6, light source; 7, porous sieve; 8, ozone generator; 8, gas cylinder.

Photochemical experiments were carried out in a laboratory-scale reactor with an effective volume of 2.5 dm^3^ (Figure 16). The heterogeneous reaction mixture (TiO_2_ suspension) was circulated by using a peristaltic pump through the reactor and the buffer vessel and by continuously bubbling air with a flow rate of 40 dm^3^·h^−1^ within the reactor. The photon flux of the internal light source (40 W, λ_max_ = 350 nm, *i.e.*, UVA range) was measured by tris(oxalato)ferrate(III) chemical actinometry [39,40]. It was estimated to be 4.3 × 10^−6^ mol photon dm^−3^·s^−1^.

### 3.3. Analytical Procedures

For analysis, 4-cm^3^ samples were taken with a syringe from the reactor through a septum. The solid phase of samples, when necessary, was removed by filtration using Millipore Millex-LCR PTFE 0.45-μm filters. The pH of the aqueous phase of the reaction mixture was measured with a SEN Tix 41 electrode.

Degradation of Triton X-100 was followed by 1290 Infinity UHPLC system (Agilent Technologies Inc., Santa Clara, CA, USA), equipped with a binary gradient pump, automatic injector, column thermostat, DAD detector and Chemstation data acquisition system. Band profiles of Triton X-100 were recorded at 223 nm. The column used during the experiments was a 100 × 2-mm Synergy HydroRP C18 (Phenomenex, Torrance, CA, USA) column packed with 2.5-μm particles. The column was thermostated at 50 °C. The eluent flow rate was 1 cm^3^·min^−1^. The composition of the mobile phase was 65:35 methanol:water for five minutes of analysis, and it was changed to 75:25 in the next five minutes.

The individually identifiable organic compounds of the liquid samples were determined by the gas chromatography-mass spectrometry method. During the sample preparation, a 4-cm^3^ solution sample was extracted with 6 cm^3^ of chloroform (Chromasolv). During the extraction, the two phases were shaken for 20 min. After the two phases separated, the extract was filtered with a 0.45-μm syringe filter and gently evaporated to dryness in a nitrogen gas flow, then re-dissolved in 60 μL of chloroform. The as-prepared samples were analyzed by gas chromatography-mass spectrometry (GC-MS). The injector was 270 °C. The separation was carried out on an Agilent 6890N gas chromatograph with an Agilent DB 5-ms UI column (30 m × 0.25 mm × 250 μm). As a detector, we used an Agilent 5973 N-type mass spectrometer in scan mode (*m*/*z* = 33–550). The ion source switched on with a 5-min delay from startup (=solvent delay). The temperature program for the separation was as follows: the initial column area was set to 60 °C and kept there for 1 min. Then, the temperature was raised to 310 °C at 8 °C/min and held for an additional 5 min. The temperature of the GC/MS interface was 280 °C. As a mobile phase, high purity helium was used with a 1 mL/min flow rate. An ~1-μL sample was injected into the column without dividing the sample flow (splitless).

The ozone concentration was determined by iodometry, using sodium iodide as the reagent and sodium thiosulfate for the titration of the iodine formed [41].

The absorption and emission spectra were recorded with a Specord S 100 diode array spectrophotometer (Analytik Jena, Jena, Germany) and a PerkinElmer LS 50B spectrofluorometer PerkinElmer, Waltham, MA, USA), respectively, using quartz cuvettes of various path lengths. Mineralization was followed by measuring the total organic carbon (TOC) concentration, by application of a Thermo Electron Corporation TOC TN 1200 apparatus (Thermo Electron Corporation, Beverly, MA, USA).

## 4. Conclusions

Triton X-100, as the most-widely applied representative of alkylphenol ethoxylate-type non-ionic surfactants, was degraded and mineralized by TiO_2_-mediated heterogeneous photocatalysis. As other advanced oxidation procedures, ozonation and treatment with peroxydisulfate were also investigated under the same conditions for comparisons. Besides, the combination of these advanced oxidation procedures (AOPs) with photocatalysis was also studied. While TiO_2_-mediated heterogeneous photocatalysis proven to be an efficient method for the mineralization of this surfactant, its combination with the other AOPs did not increase the degradation and mineralization rate. These results deviate from those observed earlier for the treatment of ionic surfactants and may be attributed to a different mechanism in which no hydroxylation of the aromatic rings takes place. Monitoring the progress of photocatalytic mineralization of the Triton X-100 components by GC-MS, both the starting tenside molecules and intermediates formed via fragmentation were followed. While cleavage of the polyethoxylate chain took place in the early stage of the photocatalytic process, the alkyl part of the tenside molecules was mineralized much more slowly.

## Supplementary Materials

Supplementary materials can be accessed at: http://www.mdpi.com/1996-1944/8/1/0231/s1.
