# Supplementary material for: TiO2-Mediated Photocatalytic Mineralization of a Non-Ionic Detergent: Comparison and Combination with Other Advanced Oxidation Procedures
Source: Materials (Basel). 2015 Jan 14;8(1):231–50. doi: 10.3390/ma8010231 (PMC5455217; doi:10.3390/ma8010231)
Supplement: Supplementary file 1 [file materials-08-00231-s001.pdf]

Supplementary Materials

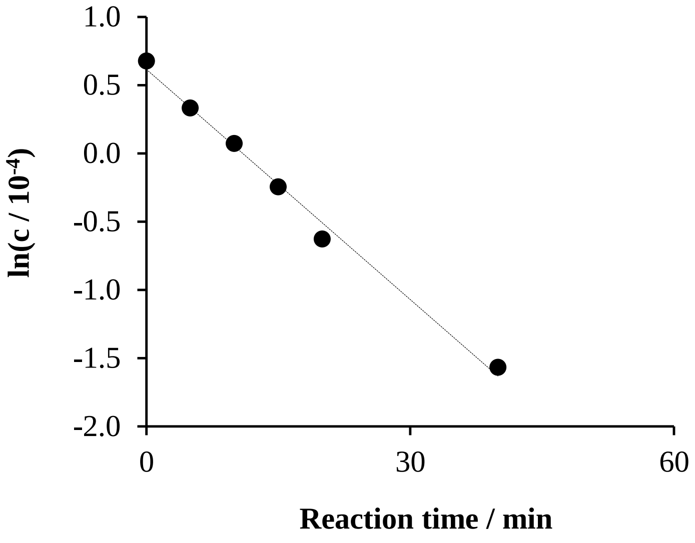

Figure S1. Ln(*c*) *vs.* time plot for the starting tensid during the photocatalysis in the aerated system containing 2 $\times 10^{-4}$ mol dm^{-3} Triton X-100 and 1 g dm^{-3} catalyst (ℓ = 1 cm).

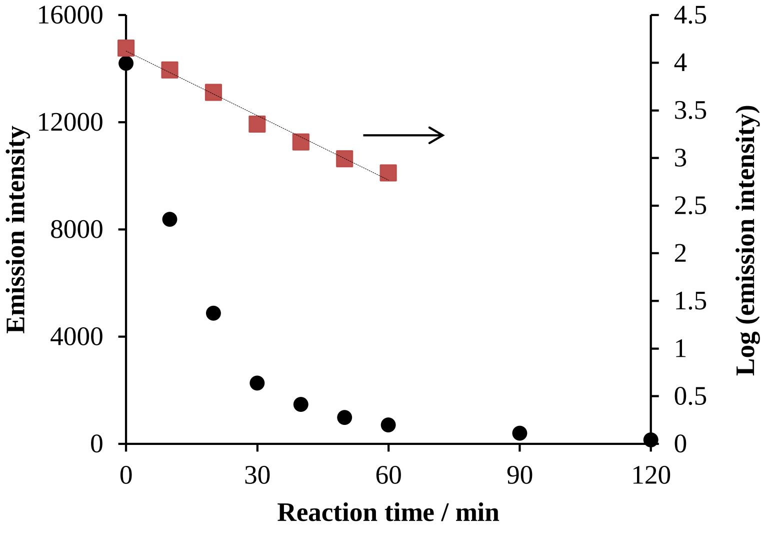

Figure S2. The change of the emission intensity (after removal of the suspended TiO_2) during the photocatalysis in the aerated system containing 2 $\times$ 10^{-4} mol dm^{-3} Triton X-100 and 1 g dm^{-3} catalyst (**l** = 1 cm, λ_{ex} =277 nm, λ_{em} =302 nm).

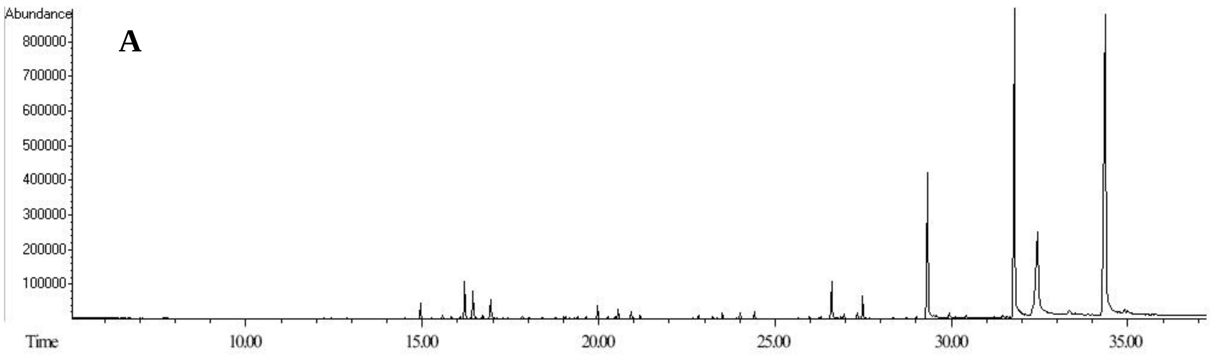

Figure S3. *Cont.*

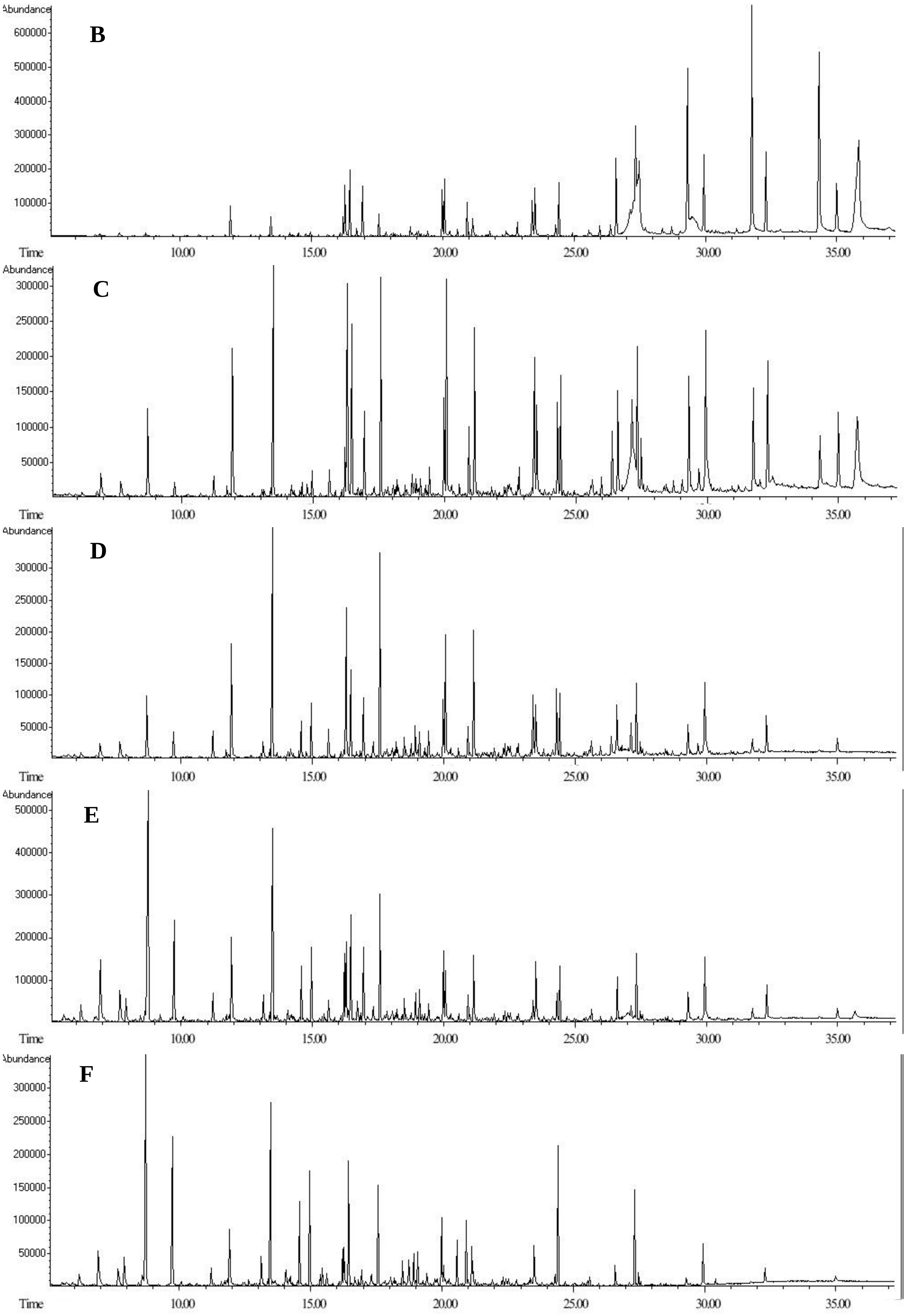

Figure S3. *Cont.*

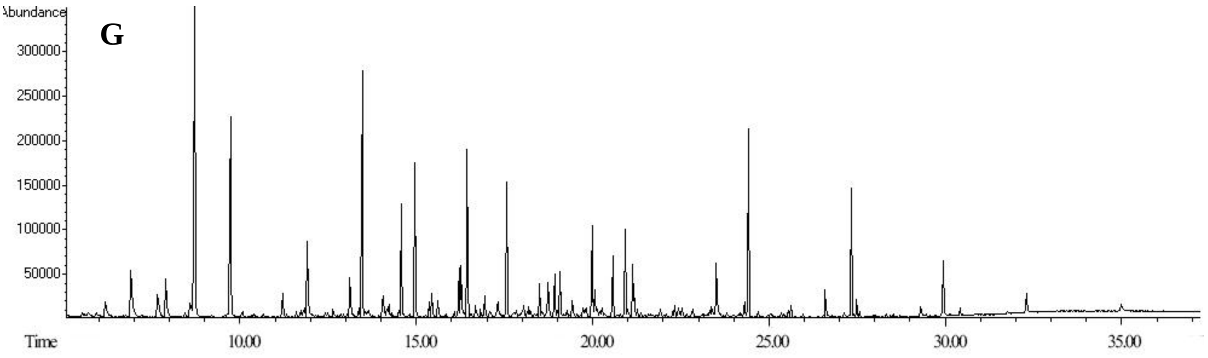

Figure S3. Total ion chromatogram of the components extracted from the reaction mixture after 0 min (**A**); 10 min (**B**); 30 min (**C**); 60 min (**D**); 90 min (**E**); 120 min (**F**); and 180 min; (**G**) irradiation.

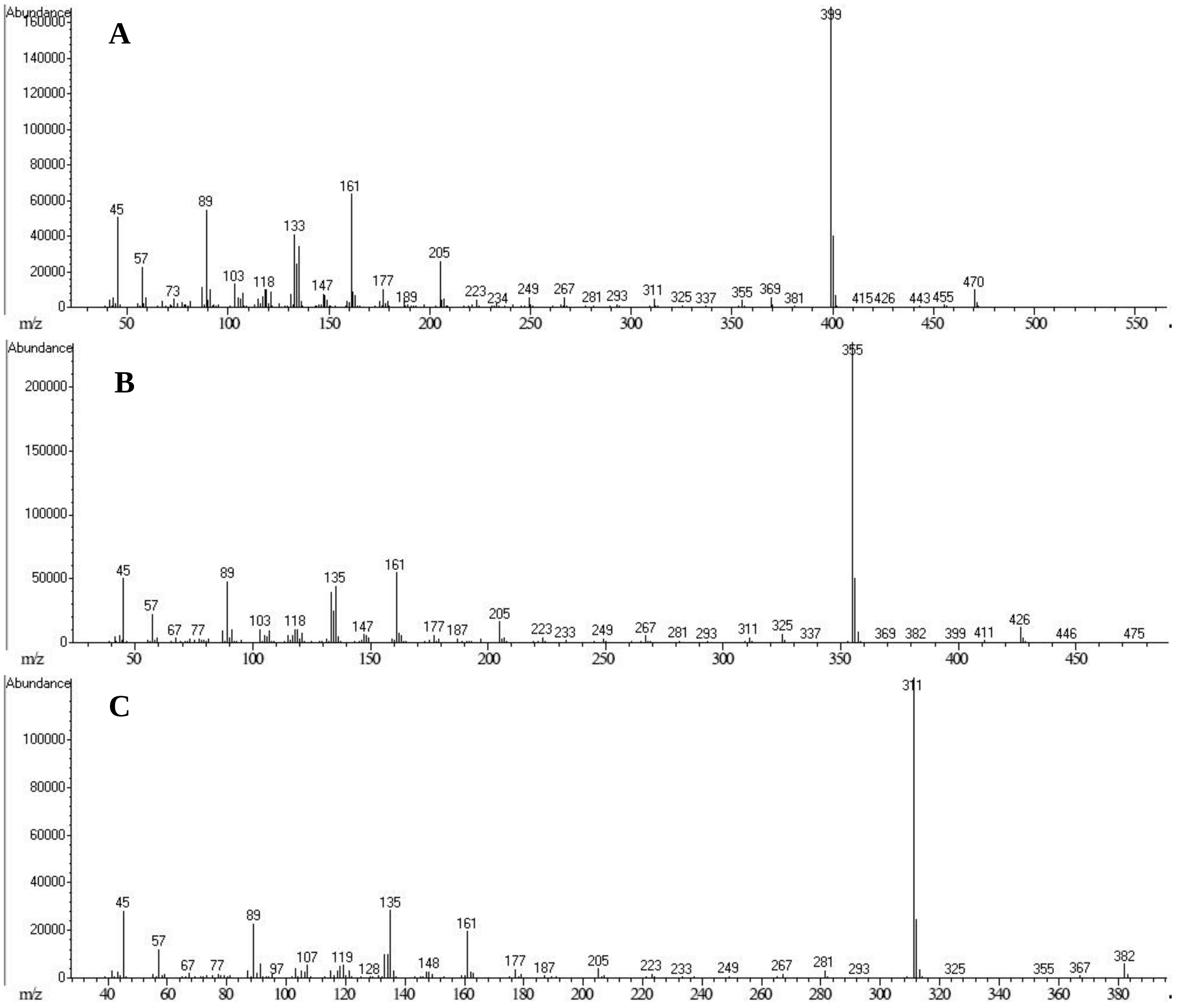

Figure S4. *Cont.*

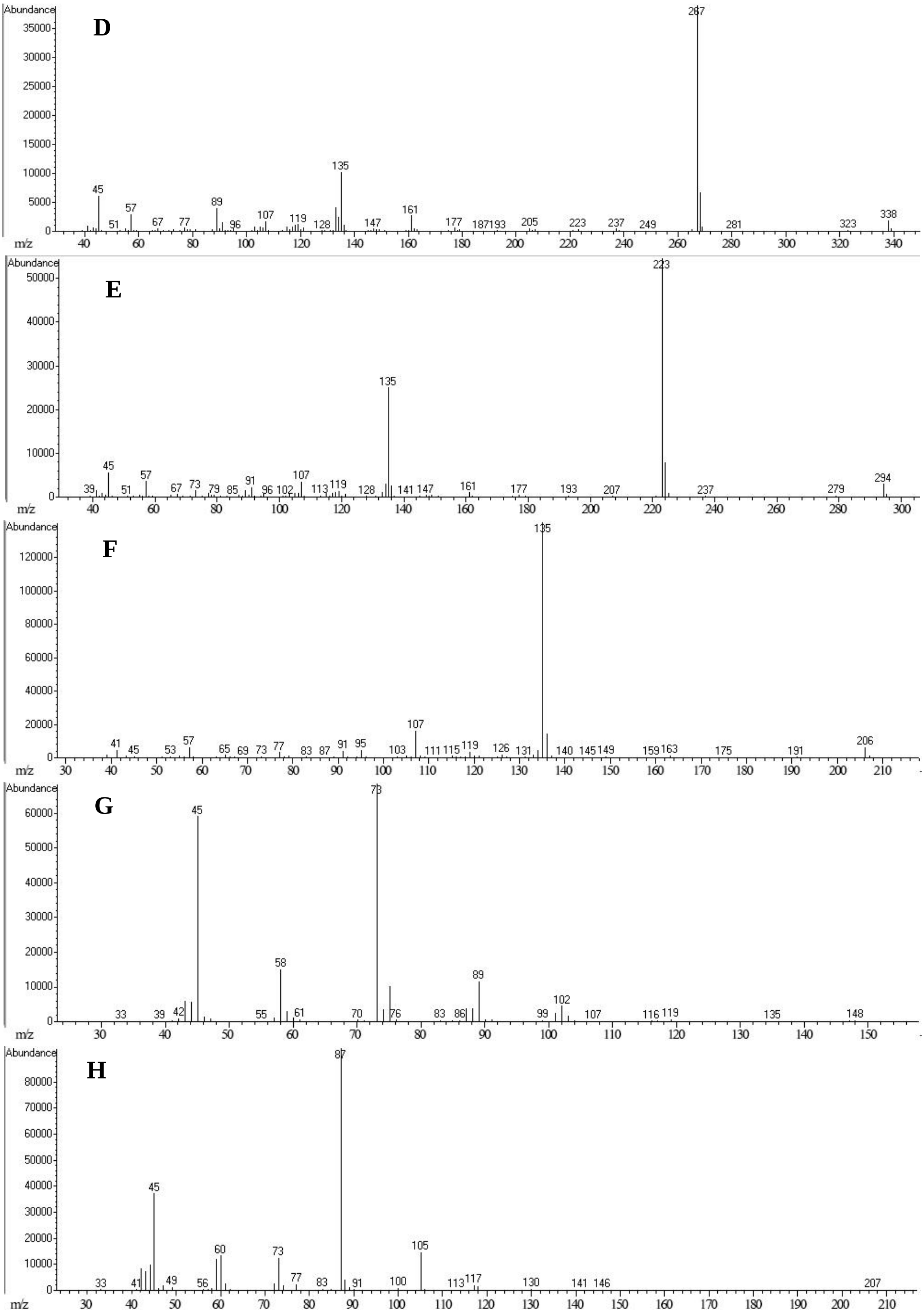

Figure S4. Mass spectra of the typical components extracted from the reaction mixture. The corresponding retention times: 34.36 min (**A**); 31.78 min (**B**); 29.32 min (**C**); 26.60 min (**D**); 23.51 min (**E**); 16.46 min (**F**); 11.92 min (**G**); 9.73 min (**H**).
